# Outline of a Non-Deliberative, Mood-Based, Theory of Action

**DOI:** 10.1007/s11406-016-9809-5

**Published:** 2017-02-07

**Authors:** Erik Ringmar

**Affiliations:** Department of Political Science, Lund University, Lund, Sweden

**Keywords:** Moods, Actions, Emotions, Freedom of the will, Intentionality, Attunement, Habit, Benjamin Libet, William James

## Abstract

In a series of famous experiments, Benjamin Libet claimed to have shown that there is no scientific basis for our commonsensical understanding of freedom of the will. The actions we are about to undertake register in our brains before they register in our conscious minds. And yet, all that Libet may have shown is that long-invoked notions such as “the will” and “freedom” are poor explanations of how actions are initiated. Actions take place as we respond to the call of the mood of the situation in which we find ourselves. Action is a way of attuning ourselves. Simple actions happen as long established habits kick in, and complex actions happen as the mood of a situation comes to correspond to the mood of a story we have been telling ourselves. When it feels right, we just act.

According to a widely accepted folk wisdom, actions are caused by consciousness.^1^ There are questions, to be sure, concerning how rational we actually are and how carefully we actually deliberate, but the commonly taken for granted assumption is that it is conscious processes in our minds which make us do one thing rather than another. Indeed, some such view seems to be required for us to be able to talk about freedom of the will and moral responsibility. Yet the widely accepted folk wisdom is rejected by neurological research. As scientific experiments conclusively have revealed, we become consciously aware of our actions only *after* they have been initiated by precognitive activity in the brain. If we accept this conclusion, and for purposes of this article we will, we must look for another way to explain action. In what follows we will provide an outline of such an alternative.

What the neurological system is reacting to, we will argue, is a mood. A mood places us in a certain situation in a certain fashion; a mood allows us to report on how we find ourselves in the world and how we feel about the way we find ourselves. The mood attunes us to a situation. Attunement, in turn, requires us to act. It is as though the mood were calling out to us and our action can be understood as an answer to this call. We are being solicited, as it were, and the action is our response to this solicitation. The interaction between a call and a response is a pre-conscious, non-deliberative, process which registers in the neurological system before it registers in our conscious minds.

## Neuroscience and Free Will

In a famous set of experiments, Benjamin Libet, a neuropsychologist at the University of California, asked subjects to flick a switch but also to tell him, by their reading of a dial, when they first had had a conscious intention to carry out the action in question (Libet 1985, 529–39). This moment in time was then compared with the moment when, according to a scan of the neurological system, the brain first gave an indication that a switch-flicking action was about to take place. The results were clear: for all subjects, the brain got ready before any conscious awareness of a decision to act — the brain reacted on average 535 ms before the muscles moved and 345 ms before the action registered in consciousness (Libet 1985, 532). From these results Libet concluded that conscious thought cannot be the cause of what we do but is instead merely registering an action which the brain already has started carrying out (Libet 1985, 536–37). These conclusions have subsequently gained wide assent among neuroscientists. To next to everyone’s satisfaction science has proven that the folk belief in freedom of will is a myth (Haggard 2008, 934–46; Wegner 2003, 65–69).

This is far from a novel conclusion (Pockett et al. 2009; Roskies 2013, 33–59). Libet’s experiments only provide better scientific evidence for a point most notoriously made by Thomas Huxley, the Victorian popularizer of Darwinian science. Animals, Huxley argued, move in response to the environment they encounter but their movements are dictated by their instincts and not by their reason (Huxley 1899, 238). As long as their movements are not constrained by outside forces, animals can move around freely, but they have no freedom of the will properly speaking and the will plays no role in determining what they do. What is true for animals, is true for human beings too. “It seems to me that in men, as in brutes, there is no proof that any state of consciousness is the cause of change in the motion of the matter of the organism” (Huxley 1899, 244). And if this is the case, it follows that

our mental conditions are simply the symbols of consciousness of the changes which takes place automatically in the organism; and that, to take an extreme illustration, the feeling we call volition is not the cause of a voluntary act, but the symbols of that state of the brain which is the immediate cause of that act. (Huxley 1899, 244)

“We are conscious automata,” Huxley concluded, and thereby “parts of the great series of causes and effects which, in unbroken continuity, composes that which is, and has been, and shall be – the sum of existence.”

This is not to say that consciousness is merely epiphenomenal. Indeed, as the French psychologist Théodule Ribot concluded in his *Les maladies de la volonté*, 1883, consciousness could still intervene to block an action (Ribot 1883, 12–24). For example: even if the ultimate cause of what we do is to be found in instincts or drives, we might still be able to control which instinct or drive that comes to be expressed in action. Our physiological urges can be channeled, directed and perhaps even stopped entirely by a conscious acts of the will (Smith 1992, 27–65; Mele 2013, 73–86). The body proposes, as it were, but consciousness disposes. To be deliberative, and to act rationally, is more than anything a matter of learning how to master our bodies and to restrain and control ourselves. The argument which Benjamin Libet is making, a hundred years later, is much the same. The only role he ascribes to consciousness is the power to veto the potential actions which the body comes up with (Libet 1985, 536–38). Consciousness is a gate-keeper of sorts, but gate-keepers do not cause things to happen, they merely direct that which is caused in other ways. Instead of a free will there is a free won’t (Roskies 2013, 47).

## Finding Ourselves in a Mood

The question to ask is obviously what those initial stirrings of the neurological system are about. If we accept that they are not the result of conscious deliberations, they must be caused in some other fashion. The argument to be developed here is that they are the result of moods. But before this argument can make sense we need to learn more about moods. There are at least three different ways in which moods have been discussed in the literature: as a matter of the affective state of an individual, as a feature of an environment, or as matter of the relationship between individuals and environments.

Consider first moods as they pertain to the affective states of individuals. Here a basic distinction is often drawn between moods and emotions (Carroll 2003, 525–533; Ratcliffe 2005, 43–46). Emotions have cognitive content whereas moods do not. Emotions are about something or someone whereas moods concern a more general attitude or stance. A mood is not an affective state which we have as much as an affective state in which we find ourselves. If you find yourself in a mood, the mood is determined outside of your conscious awareness, it organizes the world in a certain fashion or colors it in a certain hue (Carroll 2003, 529–531; Silver 2011, 207–15). Moods thus understood affect our bodies and not only our minds, and as a result they can often be interpreted already from a person’s posture, gait and general demeanor. A bored person rests her head in her hands; she is slumped on a sofa in a limp and listless position, and a depressed person is often literally pressed down by life (Straus 1952, 549). As a result the moods we are in may be obvious to others before they are obvious to ourselves. “You are in a very chipper mood today!” someone might tell us as we climb the stairs in a few sprightly steps while whistling a cheerful tune. Indeed, it may only be once others find us in a certain mood that we find ourselves there too. Our bodies are in a mood before we are and the mood in which we find ourselves influences us before we become consciously aware of it.

But we often also talk about a certain environment as having a certain mood. Here mood corresponds to what we might talk about as an “atmosphere,” understood as a spatial bearer of affect (Böhme 1993, 113–126). Atmospheres are at the same time highly elusive and perfectly obvious, and although they are difficult both to conclusively define and to firmly capture there is usually no doubt regarding their singular qualities (Anderson 2009, 78; Silver 2011, 207–215). Thus a restaurant may have a “cozy” atmosphere, a castle a “spooky” atmosphere, an Italian seaside town a “romantic” atmosphere, and so on. The atmosphere allows us to define a situation as a situation of a certain kind and it makes it possible for us to successfully navigate both physical and social obstacles or to take advantage of physical and social opportunities. Even though we may not be able to explain how it is done, we understand atmospheres automatically, often in a flash, and without much explicit ratiocination.

Yet ultimately moods are not best understood as pertaining to individuals or to a certain environment but instead to the relationship between the two (Cf. Robbins and Aydede 2008). A mood is a matter of how individuals come to adjust themselves to the situations in which they find themselves. A mood is a question of how we fit in. That is, a mood is a matter a *Stimmung*, an “attunement.” The musical metaphor is apt. When we tune an instrument we adjust its pitch to other instruments or different strings to each other. In much the same fashion, we attune ourselves by adjusting our bodies and our minds so that they correspond to the mood of a certain situation (Ratcliffe 2002, 289–290). *Das stimmt*, Germans say if they find something to be correct, and Swedes may *instämma* with a previous speaker, meaning that they agree.

How we are attuned to a situation is a question of how we feel (Ratcliffe 2005, 45–47; Rosfort and Stanghellini 2009, 256–63). If we are in tune, we will feel at home in the world and we will behave appropriately and as expected. If we are out of tune, by contrast, we will feel homeless and out of place. And obviously there are a number of intermediary stages between perfect attunement and total disconnect and also many different ways in which we can fit, or not fit, in. When somebody asks us “how are you feeling?”, we tell them — “I feel rootless,” “awkward,” “worried,” “hopeful,” “pensive,” “over the moon,” and so on. These answers provide a report on the mood we are in. That is, they report on our conscious attempt to make sense of the preconscious ways in which we fit into the situations in which we find ourselves.

Moods can help us explain a number of well-known phenomena that occur in the mysterious intermediary zone that exists between our fully conscious and fully non-conscious selves. This is where we find “gut feelings,” “sixth senses,” motor memories and feelings that are “in our bones” rather than in our minds. Although we know there is nothing to be afraid of, a scary mood will alert us to a danger; we have “eyes in the backs of our heads,” as it were, and we feel as though someone is looking at us. Or perhaps we feel as though we have been in a certain place before, or met the same person or said the same thing, although we know this cannot possibly be the case (Brown and Marsh 2010, 43). The intermediary zone is also where intuitions reside. That is, the “feeling of knowing with certitude on the basis of inadequate information and without conscious awareness of rational thinking” (Shirley and Langan-Fox 1996, 564; Cf. Dietrich 2004, 1017). We have an *Ahnung*, an inkling or a hunch; there is something that we want to say and although we cannot quite spit it out, it is right there on the “tip of our tongues” (Schwartz and Metcalfe 2011, 737–749). The existence of such *Ahnungen* provide the best retort to the snarky philosopher who insisted that

a man cannot search either for what he knows or for what he does not know. He cannot search for what he knows — since he knows it, there is no need to search — nor for what he does not know, for he does not know what to look for. (Plato 1914, 3:26)

It is in the intermediary zone between our conscious and non-conscious selves that we know things without knowing them and where we do not know the things we know. This is also where creative processes begin. Consider an artist who is working on a painting (Gendlin 1992, 348; Dietrich 2004, 1011–1026). Something is missing from the picture but it is not quite clear what. She tries a brush stroke but realizes immediately that it is the wrong one; she tries something else and this turns out to be the right way to proceed. She knows because she feels it to be right; the addition provides an appropriate attunement to the mood conveyed by the painting. The intermediary zone is a zone of emergence, we could perhaps say. It is here that things first appear, as though in a clearing in a forest (Heidegger 1993). Things come to be by making themselves known. That which comes into the light announces itself to us. As a result the new is something that we discover rather than something we invent. This, at least, is how it feels. The gut, the bones, the eyes in the back of our heads, are telling us in which direction to go and the researcher and the artist are led rather than leading.

## Moods and Simple Actions

Let us return to the question of how to account for actions. Consider first actions of Libet’s simple, finger-flicking, kind. More than anything simple motor actions such as these take place as we come to attune ourselves to the situations in which we find ourselves. The mood of the situation requires an action and we do what we do in order to fit in. We are solicited by the mood, as it were; we hear a call to which our subsequent action is the response. And just as when someone calls out to us — or when our phones ring — we feel compelled to answer and we usually do so quite automatically and without much thought. Unless a conscious reason intervenes, we are “immediately drawn to act a certain way.”

This is different from deciding to perform the activity, since in feeling immediately drawn to do something, the subject experiences no act of the will. Rather, he experiences the environment calling for a certain way of acting, and finds himself responding to the solicitation. (Silver 2011, 210–11)

This is how an empty room invites us to dance, how a path in the forest asks us to walk on it, and a newly made bed insists that we lay down (Mooney and Norris 2007, 1–9). Or consider the proverbial case of a theater in which a fire suddenly breaks out. In this state of emergency there is no time to think but luckily we do not have to. Instead of interpreting the situation we react to the mood of panic which quickly spreads throughout the building. We begin by acting, as it were, and only later will we become consciously aware of what we are doing. It is only once we already are trying to get out that we get scared (James 1884, 190). Or, to take a more serene example, consider the atmosphere prevailing in a place of religious worship. Sacred places teach not by verbal communication above all but instead by inducing a mood which draws visitors into a sense of reverence and awe. We bow our heads and pray since this, clearly, is what the situation requires. This, by the way, can explain how we come to believe in often quite outrageous dogmas. We pick up the mood before we pick up the argument, and the argument makes sense when set in the appropriate mood. Afterwards, once the mood has dissipated, we may have a hard time making sense both of our actions and our beliefs. You had to be there to understand.

The actions in which we engage here are more than anything a result of patterns of behavior which have come to be internalized by our neurological systems. They are a result of habits (James 1890a, 1:104–27; Cf. Carlisle 2014). Habits, a neuroscientist will explain, are “sequential, repetitive, motor, or cognitive behaviors elicited by external or internal triggers that, once released, can go to completion without constant conscious oversight” (Graybiel 2008, 361). Habits allow us to do something because we have done it before, and because we have done it before there is no reason to pay explicit attention to what we are doing. Our actions are offloaded to our bodies, as it were. And it is a good thing too. If we had to consciously process all the sensory information, and to explicitly monitor our bodies and our minds, we would never be able do much of anything. If we had to compute all the variables involved, we would not be able to even walk across the floor (Noë 2009, 117–20). Instead, when solicited by a mood, our reactions are instinctive and automatic. In fact, the more we think about what to do, the less insistently the mood will speak to us and the more badly attuned we will feel (Silver 2011, 210–11; Cf. Jastrow 1906, 116–39).

Compare the role of expertise. An expert is not someone who interprets a situation better or more quickly than others; instead, for the expert, the issue of interpretation does not even arise. We recognize our sister or a friend immediately and without thinking about it, and if we are asked how we know that a certain person is our wife, the answer is that “we just know” (Polanyi 1969, 124–137). Or consider the expertise that goes into the knitting of a sweater (James 1890a, 1:119). For experts, knitting is not a fully conscious activity since they can do it without thinking about it, but neither is it a fully automatic activity since we would not say that their hands are doing it by themselves. In the intermediate zone between consciousness and automaticity there is a feel for what we do, and this feel is in our fingers — as a *Fingerspitzgefühl* — more than in our minds. A well-established sequence of habitual behaviors is initiated, and once initiated, it just unfolds. It is only when something occasionally goes missing or goes wrong, when we drop a needle or a stitch, that we have to stop and pay explicit attention to what we are doing. Our response will most likely be emotional. Suddenly we feel out of place, our habits no longer apply and our expertise has been made redundant. We feel frustrated and perhaps angry (Gendlin 1973, 385). Desperately, we begin looking for a way to readjust ourselves or, alternatively, for a way to alter the situation we are in. We say a prayer in the burning theater and we utter a curse in the place of religious worship.

Simple motor actions, we can thus conclude, can be explained as attunements to a mood. It is as though we had been primed to act in a certain manner (Bargh and Chartrand 1999, 462–79; Bargh and Ferguson 2000, 925–45). The mood presents us with certain options and when it feels right we just act. Or, to be more precise, we respond to the solicitations of the mood by embarking on the initial stages of a sequence of habitual behaviors which, when awareness of the sequence eventually reaches consciousness, we already are in the midst of carrying out. The experimental situation in which Benjamin Libet placed his subjects also placed them in a certain mood we can conclude. There was a switch in their hands, a dial before them, and they were instructed to flick the switch whenever the urge came over them. Attuning themselves to this mood, their brains responded before their conscious minds had had time to react.

## Moods and Complex Actions

As several authors have pointed out, the simple motor actions that Libet investigated are not the only ones in which human beings engage. There are other, far more complex, actions which are characterized by the fact that they are consciously planned, perhaps a long time in advance. We make a plan, let us say, to go to the beach (Gallagher 2009, 115–16; Cf. Searle 1983, 79–111). Later, when we find ourselves on the beach, we explain this fact by reference to the plan we made earlier. The plan provides the reason which motivated us and the reason served as a cause of what we did. Or, in the language of intentionality, we might say that the plan contained an intention to act which we successfully went on to implement. Plans, thus understood, can always be found in the context of a narrative. We tell stories about ourselves, our lives, about the situations in which we find ourselves and the things we try to accomplish (Hutto 2008). We are the subjects of these stories and the reasons are the reasons that make sense in the context of the narrative. On a daily basis, almost minute by minute, we tell all kinds of stories to ourselves, making a wide assortment of plans which we one day, given the right circumstances, would like to implement. Our will is free, we like to think, since we can decide whether or not to put the stories into action. We can make them happen or not; we can decide to do, or not to do, what we have planned.

Consider the difference between the subject who engages in simple motor actions and the kind of subject who appears in these narratives. Perhaps we could refer to the former as an “episodic self” and to the latter as a “narrated self” (Cf. Stanghellini and Rosfort 2013, 253–55). The episodic self is the embodied self which exists from moment to moment and in each concrete situation. This is the self who is solicited by moods, who hears calls and responds to them automatically and precognitively. The episodic self is a creature of habit; it is a bundle of set routines and sequences of behaviors. The emotions felt by the episodic self are all embodied and they are nothing over and beyond their physiological expressions. The episodic self is not conscious of itself and it is has no means of self-reflection; like an animal, the episodic self is dead to itself but very good at coping with whatever situations that come up (Grosz 2013, 223; Sheets-Johnstone 2009, 290–95). The narrated self, by contrast, constantly interprets itself. The narrated self is a being whose being is an issue for itself, and it is in and through narratives that these self-reflections take place. The narrated self is a literary creation, as it were, and a figment of our imagination and of the imagination of others. It finds itself not in a situation but in a story, or rather, in a situation created by a story.

Complex actions are brought about, we might like to say, as a result of situations as they come to appear in a narrative. Complex actions are caused by the intentions that the narratives contain. But there is a problem with this argument. Stories and actions are entities of entirely different ontological kinds. Whatever happens in the story happens in our imagination, as it were, in the seclusion of our minds, but actions take place in the world and they appear before others (Arendt 1981, 92–98). In a story nothing is ever present and everything is instead always represented. In the world, conversely, everything is present — or absent or yet to be — but there is no place for representations. Because of this ontological gap there is no necessary connection between the stories we tell and the actions we perform. Narratives are too remote, as it were, too much in our heads, to make us do a particular thing rather than another or, for that matter, to do anything at all. As a result, the reasons which our stories provide cannot make us act and intentions cannot be the causes of our actions.

This is why even the most persuasive of reasons often fail to persuade. There is a difference, as W.D. Falk explains, between “having a reason” and “acting for a reason” (Falk 1963, 702–718). Having a reason to do something does not necessarily mean that we do what we do for those reasons.

What tells decisively in favor of a certain action may provide a ground for a change of attitude, and what gives a ground may become a person’s motive, but there is no contradiction involved in saying that that which gave a ground failed to motivate in any way. The concept of a “ground” implies nothing more than that known grounds are available to supply guidance to someone who is willing to take guidance from them. (Falk 1963, 709)

What intervenes here and stops the representation from presenting itself is the fact that human beings always find themselves in situations to which they must attune themselves. The presence of the world is always far more pressing than the representation provided by the story. The reasons contained in the story were indeed very good ones but somehow or another we did not think of them when it mattered. We meant to do it, but it did not feel right at the time; we got lazy, made up pretexts, had to wash our hair and walk the dog. The mood never solicited us, or we ignored the solicitation. Since we never got, or heard, the call, there is nothing to which we have to respond.

A number of curious facts can be explained in this fashion. Take akrasia — the classical problem of why “I do not do what I want, but I do the very thing I hate.” Curiously, there seem to be two wills within the same person, or perhaps multiple persons fighting it out between themselves. But it makes more sense to understand this as a conflict between the plans made by the narrated self and the mood of the situation in which the episodic self finds itself. It is when the mood of a situation solicits us and when we automatically respond to that mood that the bad old habits kick in. “For of a perverse will comes lust,” as Saint Augustine put it when contemplating this conundrum, “and a lust served becomes custom; and custom not resisted becomes necessity” (Saint Augustine 1867, 164; Cf. Carlisle 2014, 113–19). This is how the young Augustine became a sinner. But we find the same conflict whenever we defer to an external authority and abandon or postpone our plans. Despite our better judgment we succumb to the rhetoric of the political orator, are carried away by the enthusiasm of the crowd and somnambulistically imitate what everyone else is doing (Tarde 1895, 213–396). In these cases and others like them, we lose ourselves in the mood created by the situation. We are no longer ourselves; that is, we are no longer the narrated selves that appear in the stories we tell about ourselves.

## How Narratives Are Called by Moods

In order to explain complex actions, we need to find a cause which is more proximate than the stories we tell. We need to find a cause which has the same ontological status as the actions themselves. When W. D. Falk wrote about this problem, he identified the importance of what he called “minding” (Falk 1963, 711–12). In order for a reason to serve as a cause, he argued, we must “mind” the reason in question, not in general, but right at the time and place when it matters. We may, for example, have a good reason not to pour ourselves another drink, but unless we mind that reason when we already have had one drink too many, we may still reach for the bottle. Falk’s minding, let us suggest, provides the kind of causal factor we are looking for. Minding is something present, not something represented in a story. Yet, arguably, the term itself needs to be replaced. “Minding” is, well, too much in the mind. An alternative phrase which Falk uses is “taken to heart” — a reason, he says, “must be not only known but also ‘taken to heart’” — meaning, “keenly felt” or “greatly affected by” (Falk 1963, 705). What Falk refers to is the interaction that takes place between the person — mind and body — and the situation in which she finds herself. Perhaps we could simply say that minding is a matter of attunement.

A first thing to notice here is that narratives — much as the *Ahnungen*, hunches and intuitions we discussed above — suddenly just appear. We are not coming to the narrative but instead the narrative seems to be coming to us. “Ideas occur to us,” Max Weber once noted, “when they please, not when it pleases us” (Weber 1946, 136). Compare dreams where associational linkages between images and memories quite automatically are strung together and given a narrative connection (Montangero 2012, 159; Cf. Ringmar 2016). Since we are not awake, we cannot control these narratives — and the connections as a result have no proper beginnings, climaxes or conclusions — instead the story-telling happens as if by itself and it is not dependent on the presence of a self-conscious narrator. The way the dream appears to us is replicated by the way narratives continuously appear in our daily, fully-awoken, lives. Suddenly a thought, and idea or a poem just pops up; it is as though the Muses were talking to us; as though we were given a message by an angel or a god.

What is curious about these unexpected gifts, Weber went on to explain, is that they are given “not when we are brooding and searching at our desks,” but instead when we leave our work behind and engage in some entirely different activity, such as “when smoking a cigar on the sofa” or “talking a walk on a slowly ascending street” (Weber 1946, 136; cf. Dietrich 2004, 1019–20). Thoughts, that is, appear more easily when we find ourselves in some moods rather than in some others. Thus it might be easier to engage in creative writing in a café, or perhaps in the restaurant-car of a train, than to write at home, or perhaps we might find that deserts are particularly good places to contemplate spiritual matters (Jasper 2008). The reason that narratives come to us here is that narratives too have moods. The mood of a story as a whole is often defined already by its genre, but each scene which the story contains has distinct moods of its own. Compare a movie where the accompanying sound track sets the mood of each scene and in that way explains to the audience what they can expect to happen (Cohen 2010, 879–908).

For a narrative to come, the mood of the situation in which we find ourselves must solicit the mood of the narrative. This is what happened when Weber walked along that “slowly ascending street.” After all, to walk upwards in this fashion is likely to put us in a certain mood. It requires some effort, but as long as the street is only slowly ascending, the effort is not too great. Our legs seem to be forcing our thoughts; the mood is making our thoughts move onwards and upwards. The story that comes is a story whose mood fits with the mood of the situation we are in.

In many cases it would rather seem to be the change of moods which solicits the narrative. As Weber noticed, our best thoughts often come to us when relaxing after a period of intense concentration, yet, as he went on to say, the ideas would not come unless we first had prepared for their arrival. Compare the way the philandering Augustine finally turned into a saint (Saint Augustine 1867, 203–4). As his *Confessions* make clear, he only abandoned his life of debauchery when he, one day, laid down to weep under a fig-tree. Suddenly, he heard a voice calling out to him: “Take up and read; Take up and read,” and this was then he picked up the Gospel. The situation was calling out to him and he responded but only because his years of contrition had prepared him to do so. Like Augustine, we can conclude, we must make ourselves “ready for the readiness of holding oneself open for the arrival, or for the absence of a god” (Heidegger 2009, 58).

The moods which Weber described called for thought; Augustine’s mood called for action; yet both thoughts and actions are ways of attuning ourselves to the situations in which we find ourselves. In simple motor actions, this call and response is automatic and precognitive. The solicitation can be answered by embarking on the initial stages of a sequence of habitual behaviors which, when awareness of the sequence eventually reaches consciousness, we already are in the midst of carrying out. However, if we find ourselves in a situation with which our habits cannot deal appropriately, we instead have to stop, to think or act. The episodic self must call upon the narrated self and the narrated self must respond to this call (Dewey 1922, 75–76).

When relaxing after a period of intense concentration, there may be nothing much that demands an action. We can let our thoughts soar, going off in various directions, exploring connections which we never thought of before. But other moods are far more demanding. The situation may present us with a mood of unease, of dissatisfaction or perhaps with an opportunity. Moods such as these have directions, as it were, and they have implications. This directionality is a fully embodied sensation (Gendlin 1992, 341–353). The unease might feel like a heaviness in our chest, the dissatisfaction like a small cramp in our gut or a numbness in an arm, and the opportunity like an itch or like a faint smell. Yes, we eventually ended up at the beach, and the reason is of course that we had made a plan to go there, expressed in a narrative which featured our intention. Yet it was the beautiful weather which put us in the mood to actually go; or perhaps it was the mood in which we were placed by a bored, nagging, child, or the mood of anticipation in which we were found ourselves after the sudden realization that a certain person, with whom we had such fun last time we went, might be there again today. When the mood called, we acted.

## William James gets out of bed

In a series of famous experiments, Benjamin Libet claimed to have shown that there is no scientific basis for our folkloric understanding of freedom of the will. The actions we are about to undertake register in our brains before they register in our conscious minds. Consciousness arrives too late, as it were, to be included in the chain of causes. And yet, all that Libet may have shown is that long-cherished notions such as “the will” and “freedom” are poor explanations of how actions are initiated. Instead actions of both a simple and a complex kind are the result of solicitations by the moods of the situations in which we find ourselves. Simple motor actions take place as sequences of habitual movements are solicited and complex actions happen as the story we have been telling ourselves is evoked. When it feels right, we just act. In the end human beings engage in both simple and complex actions and we are simultaneously both episodic and narrated selves. We exist both concretely and fictionally; we are present but also represented; we are real but in an imaginary way. The episodic and narrated selves come together in the life that we end up living.

This is how William James finally got out of bed. It was a cold winter’s morning, he tells us, but he was perfectly comfortable under his blanket (James 1890b, 2:524–525). He obviously planned to get up — he had lectures to give and research papers to write. He knew he had to get up and he knew that he eventually would get up, and yet somehow he could not make himself do it. Then suddenly, and much to his surprise, he found that he was out of bed and that the daily routine had begun. The story which James told himself while still in bed contained a plan, we can conclude, and it contained an intention, but this by itself did not make him do what he eventually did. What made the difference was instead a change of moods. The mood was suddenly transformed from comfortable to uneasy and eventually the whole situation became quite unbearable. It was his body and not his deliberative faculties that reacted to this change. “A fortunate lapse of consciousness occurs; we forget both the warmth and the cold; we fall into some revery connected with the day’s life, in the course of which the idea flashes across us, ‘Hollo! I must lie here no longer.” The mood of the situation suddenly felt entirely differently and this is what made him act. “This case,” James concluded once he firmly and conclusively had risen, “seems to me to contain in miniature form the data for an entire psychology of volition” (James 1890b, 2:525).

## Footnotes

1 I am grateful to Peter Baehr, Christian Borch, Dana Kaplan, Janice Bially Mattern, Dan Silver and anonymous reviewers for comments on a previous version of this article. See also (Ringmar 2017). I hope to develop this argument further in (Ringmar 2018)
